# Population trends in emergency cancer diagnoses: The role of changing patient case-mix

**DOI:** 10.1016/j.canep.2019.101574

**Published:** 2019-12

**Authors:** A. Herbert, S. Winters, S. McPhail, L. Elliss-Brookes, G. Lyratzopoulos, G.A. Abel

**Affiliations:** a MRC Integrative Epidemiology Unit Bristol Medical School University of Bristol Bristol UK; b Epidemiology of Cancer and Healthcare Outcomes (ECHO) Group, Research Department of Behavioural Science and Health, University College London, 1-19 Torrington Place, London, UK; c National Cancer Registration and Analysis Service (NCRAS), Public Health England, 6th Floor, Wellington House, 135-155 Waterloo Road, London, UK; d Cambridge Centre for Health Services Research, University of Cambridge Institute of Public Health, Forvie Site, Cambridge, UK; e University of Exeter Medical School, Exeter, UK

**Keywords:** Diagnosis, Emergency, Neoplasm, Inequalities, Public health, Case-mix

## Abstract

**Background:**

Diagnosis of cancer through an emergency presentation is associated with worse clinical and patient experience outcomes. The proportion of patients with cancer who are diagnosed through emergency presentations has consequently been introduced as a routine cancer surveillance measure in England. Welcome reductions in this metric have been reported over more than a decade but whether reductions reflect true changes in how patients are diagnosed rather than the changing case-mix of incident cohorts in unknown.

**Methods:**

We analysed ‘Routes to Diagnosis’ data on cancer patients (2006–2015) and used logistic regression modelling to determine the contribution of changes in four case-mix variables (sex, age, deprivation, cancer site) to time-trends in emergency presentations.

**Results:**

Between 2006 and 2015 there was an absolute 4.7 percentage point reduction in emergency presentations (23.8%–19.2%). Changing distributions of the four case-mix variables explained 19.0% of this reduction, leaving 81.0% unexplained. Changes in cancer site case-mix alone explained 16.0% of the total reduction.

**Conclusion:**

Changes in case-mix (particularly that of cancer sites) account for about a fifth of the overall reduction in emergency presentations. This would support the use of adjustment/standardisation of reported statistics to support their interpretation and help appreciate the influence of case-mix, particularly regarding cancer sites with changing incidence. However, most of the reduction in emergency presentations remains unaccounted for, and likely reflects genuine changes during the study period in how patients were being diagnosed.

## Highlights

• During 2006–2015, emergency presentations decreased by 4.7% (23.8%–19.2%).
• Change in case-mix variables explained 19% of the reduction, leaving 81% unexplained.
• Changes in cancer site case-mix alone explained 16% of the total reduction.
• Most of the reduction likely reflects genuine changes in how patients are diagnosed.

## 1 Introduction

Evidence from several countries (including England, the US, Canada, Sweden and France) documents that substantial proportions of cancer patients are diagnosed in an emergency context [1,2], and that this patient group experiences poorer clinical outcomes compared to patients diagnosed through other routes [3,4]. Consequently in England, population statistics on emergency cancer diagnoses are used as routine measures for cancer surveillance [5].

Notable reductions in the proportion of Emergency Presentations (EPs) among incident cancer cases have been reported in England [6]. Some of these reductions may reflect improvements in diagnostic pathways, but others could reflect changes over time in patient case-mix [1]. Cancer site and socio-demographic variables are strongly associated with the risk of diagnosis through an EP [7], and the case-mix of incident cases is shifting. For example, prostate and lung cancer, which are associated with either lower risk (prostate) or higher risk (lung) of EP compared with other cancers, have contrasting incidence trends (increasing for prostate; decreasing for lung) [5]. Considering only those two cancer sites, one would expect a net reduction in the overall proportion of EPs simply due to the changes in incidence, however when considering all cancer sites and demographic factors the situation is less clear. There is currently no formal evidence to describe the degree by which changes in patient case-mix account for the observed decrease in EP.

Therefore, we aimed to examine the potential influence of case-mix changes on EP time-trends, to help understand their potential contribution and inform public reporting conventions.

## 2 Methods

### 2.1 Data

We analysed data on incident cancer patients 2006–2015, resident in England. The National Cancer Registration and Analysis Service of Public Health England assigns a diagnostic route (including EP) to all cases, using an algorithmic rules-based approach applied to cancer registration, Hospital Episode Statistics, Cancer Waiting Times, and NHS screening programme data [3]. EP is defined as diagnosis of cancer following an A&E attendance, emergency hospital admission, emergency between-hospital transfer, or emergency GP referral. Information was available on year (of diagnosis), sex, age group, deprivation group (based on the Income Domain of the Index of Multiple Deprivation of the Local Super Output Area of patient’s residence), and cancer site (35 common and rarer cancer sites, defined by ICD-10 codes: **Appendix 1**).

### 2.2 Analysis

We first described associations between four case-mix variables (sex, age, deprivation, cancer site) and diagnosis year with risk of EP, and the distribution of these case-mix variables over time.

Our general analytical approach was to fit binary logistic regression models (outcome: EP status; independent main effect variables: diagnosis year  +  all four case-mix variables). Given prior evidence, interactions between each socio-demographic variable and cancer site were also included [7]. To examine the global influence of changes over time in respect of all four case-mix variables, we used the model to predict proportions of EPs per year, had the sex-age-deprivation-cancer site case-mix remained the same as for 2006.

To examine the contribution of each case-mix variable individually, we used four simpler models, based on each variable alone (independent variables: year + one case-mix variable, e.g. sex) and predicted the expected proportions of EP if case-mix had not changed.

Lastly, to examine the unique contribution of changes in the relative frequency of each of the 35 cancer sites, we used a separate model per cancer site (i.e. 35 models). In each instance, all incident cases of cancer (not just those with the cancer of interest) were included in the model (independent variables: year + cancer site of interest [yes/no]).

## 3 Results

**Fig. 1:**
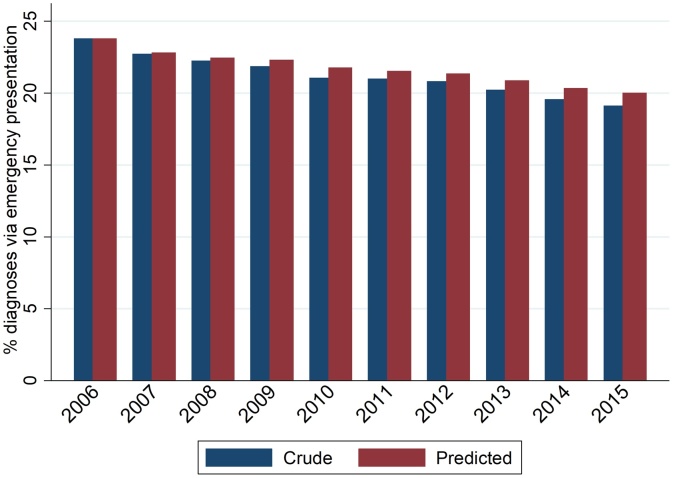
Crude and predicted* proportions of emergency presentation by year of diagnosis. *Had sex, age group, deprivation group, and cancer site case-mix stayed the same as in 2006. Predictions made using a logistic regression model fitted to 2006-15 data, where emergency presentation was the outcome, and independent variables were diagnosis year, sex, age group, deprivation group, cancer site, and interaction terms sex*cancer site, age group*cancer site, and deprivation*cancer site.

There were 2,641,428 incident cases of the 35 studied cancer sites during the 10-year study period, of which 559,254 (21.2%) were diagnosed as emergencies, decreasing from 23.8% in 2006 to 19.2% in 2015 (Fig. 1; **Appendix 2**). Given a monotonic decrease in crude proportions over time, we focus on the earliest and latest study years (2006 and 2015).

The risk of EP varied largely by age, deprivation, and cancer site, with relatively little variation by sex (**Appendix 2**). Risks increased notably with increasing age and deprivation. Melanoma, breast and prostate cancer had relatively low risk of EP, and lung and colon cancer relatively high. Compared to cancer patients diagnosed in 2006, those diagnosed in 2015 tended to be younger (51.3% in 2015 aged 70 and over vs 50.5% in 2006) and less deprived (**Appendix 3**). The relative frequency of prostate cancer or melanoma cancer increased, and that of breast or lung cancer decreased.

Between 2006 and 2015 the observed proportion of EPs decreased by 4.7 percentage points (i.e. 23.81% to 19.15%). Had the distribution of all four case-mix variables in 2015 been that of 2006, the reduction would have instead been 3.8 percentage points (Table 1). That is, changes in the distribution of sex-age-deprivation-cancer site case-mix explained 19% of the observed decrease (0.9 of the 4.7 percentage points). Alternatively, 81.0% of the reduction was *not* explained by these changes.

**Table 1:** Crude and predicted proportions of emergency presentations in 2015 (had the patient case-mix [overall and by individual case-mix variable] remained the same as in 2006).

	Crude (observed) % of emergency presentations	Predicted based on all case-mix variables*	Predicted based only on sex**	Predicted based only on age**	Predicted based only on deprivation**^,^^	Predicted based only on cancer site**
2006 (N = 237,799)	23.81	23.81	23.81	23.81	23.81	23.81
2015 (N = 285,660)	19.15	20.04	19.14	19.23	19.33	19.90
Absolute change in % between 2006 and 2015†	−4.66	−3.77	−4.67	−4.58	−4.48	−3.91
% of the crude absolute reduction that is explained by case-mix changes‡	.	19.04	−0.19	1.78	3.85	16.01

* Had sex, age group, deprivation group, and cancer site case-mix stayed the same as in 2006. Predictions made using a logistic regression model fitted to 2006-15 data, where emergency presentation was the outcome, and independent variables were year, sex, age group, deprivation group, and cancer site, and interaction terms for sex*cancer site, age group*cancer site, and deprivation group*cancer site.

** Had sex (or age group, deprivation group, or cancer site) case-mix stayed the same as in 2006. Predictions made using a logistic regression model fitted to 2006-15 data, where emergency presentation was the outcome, and independent variables were year and sex (or age group, deprivation group, or cancer site).

† Calculated as c–d where c = Crude (or Predicted) proportion in 2015 and d = Crude (or Predicted) proportion in 2006. Calculations based on proportions to five decimal places. E.g. For predicted proportions where all case-mix stayed the same throughout study period, 20.03882–23.81171=--3.77289.

‡ Calculated as 100(e–f)/e, where e = Crude proportion in 2015-Crude proportion in 2006, and f = Predicted proportion in 2015-Predicted proportion in 2006. Calculations based on proportions to five decimal places. E.g. For all case-mix, 100((19.15162–23.81171)-(20.03882–23.81171))/(19.15162–23.81171) = 19.03826.

^ The 2007 version of Index of Multiple Deprivation was used for diagnosis year 2006, 2010 version for diagnosis years 2007–2009, and the 2015 version for diagnosis years 2010–2015.

Regarding individual contributions of each case-mix variable, changes in the distribution of sex or age explained little of the above reduction in proportion of EPs (–0.2% and 1.8%, respectively, Table 1). [Negative contributions, such as for sex, indicates that had the sex distribution been the same in 2015 as in 2006, the proportion of EPs in 2015 would have been even *lower* than that observed]. Changes in deprivation case-mix explained 3.9% of the total reduction, while changes in cancer site case-mix had the strongest influence by far, explaining 16.0%.

**Fig. 2:**
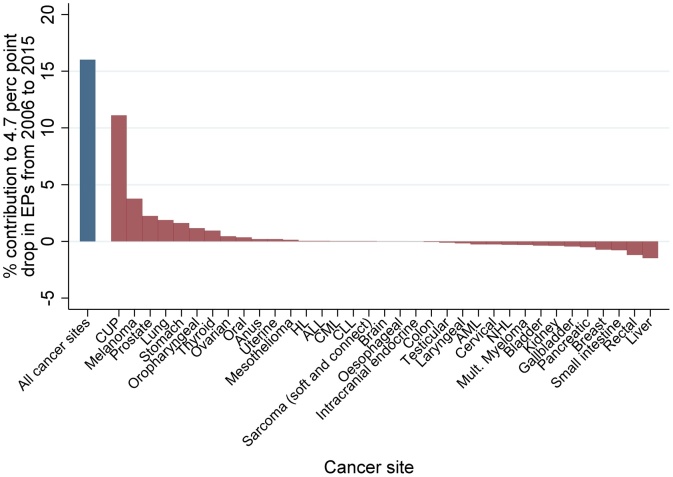
Relative proportion* of observed absolute decrease (between 2006 and 2015) in EPs attributable to changes in cancer site case-mix (overall [blue bar] and individual cancer sites [red bars]). (For interpretation of the references to colour in this figure legend, the reader is referred to the web version of this article). *Had sex, age group, deprivation group, and cancer site case-mix stayed the same as in 2006. Predictions made using a logistic regression model fitted to 2006-15 data, where emergency presentation was the outcome, and independent variables were: year and a) in the case of all cancer sites, a variable for cancer site; b) in the case of each individual cancer site, a dummy variable representing that particular site. ALL = Acute Lymphoblastic Leukaemia; AML = Acute Myeloid Leukaemia; CLL = Chronic Lymphocytic Leukaemia; CML = Chronic Myeloid Leukaemia; CUP = Cancer of Unknown Primary; HL = Hodgkin Lymphoma; NHL = Non-Hodgkin Lymphoma.

**Fig. 3:**
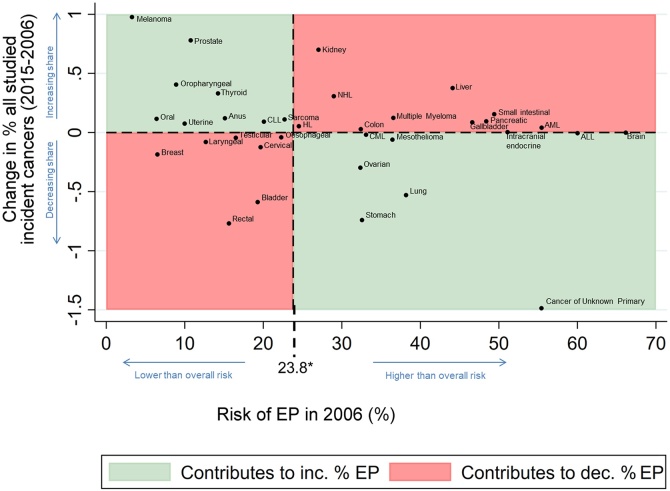
Change in the relative frequency of each studied cancer-site (between 2006 and 2015) plotted against the cancer site-specific risk of EP (in 2006). Certain cancers with relatively low risk (e.g. melanoma) are becoming more common (top-left quadrant), while some with relatively high risk less common (lung, bottom-right quadrant). All other factors being equal, these would contribute to downward trends. Contrasting patterns are observed in, for example, rectal and liver cancer (bottom-left and top-right quadrants). These observations help to further interpret and contextualise the findings, and should be interpreted together with Appendix 6 and estimates from the main analysis model, indicating that the ‘net’ (overall) effect of cancer site case-mix changes over time contributes to decreasing trends. *The overall risk of EP, across the 35 cancers studied. ALL = Acute Lymphoblastic Leukaemia; AML = Acute Myeloid Leukaemia; CLL = Chronic Lymphocytic Leukaemia; CML = Chronic Myeloid Leukaemia; EP = Emergency presentation; HL = Hodgkin Lymphoma; NHL = Non-Hodgkin Lymphoma.

The specific contribution of each cancer site to the observed decrease in EPs is detailed in Fig. 2. Cancer of unknown primary was by far the biggest individual contributor (11.1% of the observed decrease), followed by melanoma (3.8%), prostate (2.2%), and lung (1.9%). Small intestinal, rectal, and liver cancers had negative contributions of –1.5% to –0.8%. Fig. 3 shows whether each cancer site is associated with a relatively low/high risk of emergency presentation (X-axis), and whether its relatively frequency among the studied cancer sites increased/decreased during 2006-15 (Y-axis). In general, the decreasing EP trend due to changing case mix was partly explained by increased relative frequency of lower-risk cancers for EP (e.g. melanoma, top-left quadrant) combined with decreased relative frequency of higher-risk cancers (e.g. lung, bottom-right quadrant), though was tempered by increased relative frequency of higher-risk cancers (e.g. liver, top-right quadrant) and decreased relative frequency of lower-risk cancers (e.g. breast, bottom-left quadrant).

## 4 Discussion

A fifth of the reduction in the percentage of cancer patients diagnosed through emergency presentations during 2006–2015 reflects changes in patient case-mix, particularly the changing distribution of different cancer sites. Up to four-fifths of the decrease could reflect genuine changes in how patients are diagnosed as it is not explained by changes over time in key patient characteristics.

Crude proportions of emergency presentations reflect the burden of emergency presentations in the population, which is the appropriate information to be used for planning and administration of health care services. Using time-trends in these crude counts for assessing improvement in diagnostic pathways is however problematic, because they can be impacted by changes over time in case-mix. Case-mix adjusted proportions capture changes over time in the proportion of emergency presentations, having removed the effect of changes in the distribution of case-mix variables (i.e. regarding the cancer site, age, sex and deprivation of incident cases). Further, by comparing crude and case-mix adjusted estimates at the end of the study period, we can appreciate the degree by which changes can be attributed to intrinsic (i.e. case-mix) or extraneous (e.g. how patients are diagnosed) changes.

The findings build on prior inquiries on time-trends in emergency presentations [8], for the first time exploring the contributions of changes in case-mix. We have previously shown that for most of the 35 different cancer sites there were decreasing proportions of patients diagnosed through emergency presentations over time, though this was not the case for 5 cancers (laryngeal, HL, anal, AML, ALL) [8]. Expectedly given these earlier findings, these 5 cancers tend to have a ‘neutral’ (neither positive nor negative) contribution as indicated by their appearance in the ‘middle’ of Fig. 2. These considerations further highlight the usefulness of considering individual cancer site and other case-mix variable influences in trends in emergency presentations, and also highlight that new early diagnosis strategies could be particularly useful for some of these cancers. We used a large national dataset spanning ten years, and EP status was derived via robust and stable methods. Though we explored key case-mix variables, there may be residual confounding by other socio-demographic variables (e.g. ethnicity); however, the potential contribution of such variables above and beyond that of age-sex-deprivation-cancer site is likely limited.

The findings with regard to Cancer of Unknown Primary (CUP) deserve special mention, as this patient group typically represents cases of advanced stage and has a high proportion of emergency presentations (3rd highest among all 35 studied cancers, Supplementary Material Appendix 2). Given its decreasing incidence, it is likely that in later years, cases which would have been classified as CUP in earlier years, would have been assigned to a known primary group (e.g. lung cancer, colon cancer etc.). Thus, there is a gradual though subtle enrichment of each cancer site (other than CUP) with tumours more likely to be diagnosed as emergencies, as each site is progressively incorporating a small proportion of ‘ex-CUP’ cases, meaning that the overall contribution of cancer site to the reduction in proportion EP over time has been underestimated.

After adjustment for other case-mix variables (including cancer site), we have observed a relatively small contribution of deprivation case-mix differences in changes over time. This finding should be interpreted as denoting relatively fewer cases of cancer being diagnosed in more deprived groups progressively during the study period. This should not be mis-interpreted as indicating that deprivation exerts no risk on emergency presentation – it does indeed do so, and related inequalities are large and persistent [7,8].

Consistent with reporting conventions for other cancer surveillance measures such as incidence or survival, routine surveillance statistics on emergency presentations should, alongside crude estimates, account for the influence of changing case-mix (such as by providing case-mix adjusted/standardised estimates). This can support interpretation and help appreciate the influence of case-mix, particularly regarding cancer sites with changing incidence [9,10].

To conclude, whilst changing case-mix has an influence, the vast majority of the reduction in the percentage of cancer patients diagnosed through an emergency presentation likely reflects changes in how patients are diagnosed. These changes have probably arisen from a combination of patients presenting earlier and better diagnostic processes post-presentation such as the use of the fast-track referral pathway [3,11,12].

## Authorship contributorship statement

Concept and Methods. GAA, AH, GL.

Analysis: AH and SW, based on foundation work by SW, SMc, LEB

Drafting: All authors.

Critical review: All authors.

## Declaration of Competing Interest

The authors have no conflicts of interest to declare.
