# Supplementary material for: Population trends in emergency cancer diagnoses: The role of changing patient case-mix
Source: Cancer Epidemiol. 2019 Dec;63:101574. doi: 10.1016/j.canep.2019.101574 (PMC6905147; doi:10.1016/j.canep.2019.101574)
Supplement: Supplementary file 1 [file mmc1.docx]

**ONLINE APPENDICES**

**Appendix 1: ICD-10 codes used to define cancer sites in this study**

| **Cancer site** | **ICD-10 code** |
| --- | --- |
| Acute lymphoblastic leukaemia (ALL) | C21.0-C21.2, C21.8 |
| Acute myeloid leukaemia (AML) | C92.0, C92.4-C92.5, C93.0, C94.0, C94.2 |
| Anal | C21.0-C21.2, C21.8 |
| Bladder | C67.0-C67.9 |
| Brain | C71.0-9 |
| Breast | C50.0-C50.6, C50.8-C50.9 |
| Cancer of Unknown Primary | C77.0-C77.5, C77.8-C78.8, C79.0-C79.8, C80.0, C80.9 |
| Cervix | C53.0- C53.1, C53.8-C53.9 |
| Chronic lymphocytic leukaemia (CLL) | C91.1 |
| Chronic myeloid leukaemia (CML) | C92.1 |
| Colon | C18.0-C18.9 |
| Gallbladder | C23.0 |
| Hodgkin lymphoma (HL) | C81.0-C813, C817, C819 |
| Intracranial endocrine | C75.1-C75.3 |
| Kidney | C64.0 |
| Laryngeal | C32.0-C32.3, C32.8-C32.9 |
| Liver (excluding intrahepatic bile duct) | C22.0, C222- C224, C227, C229 |
| Lung | C33.0, C340- C343, C348- C349 |
| Melanoma | C43.0- C43.9 |
| Mesothelioma | C45.0-C45.2, C45.7, C45.9 |
| Multiple myeloma | C90.0- C90.2 |
| Non-Hodgkin lymphoma (NHL) | C82.0-C82.2, C82.7, C82.9-C84.5, C85.0- C85.1, C85.7, C85.9 |
| Oesophageal | C15.0-C15.5, C15.8-C15.9 |
| Oral | C02.0-C02.4, C02.8-C03.1, C03.9-C04.1, C04.8-C04.9, C06.0-C06.2, C06.8-C06.9 |
| Oropharyngeal | C01.0, C09.0-C09.1, C09.8-C10.4 |
| Ovarian | C56.0, C57.0-C57.4, C57.7-C57.9 |
| Pancreatic | C25.0-C25.4, C25.7-C25.9 |
| Prostate | C61.0 |
| Rectal | C19.0-C20.0 |
| Sarcoma: connective and soft tissue | C48.0-C48.3, C48.8, C49.0-C49.6, C49.8-C49.9 |
| Small Intestinal | C17.0-C17.3, C17.8-C17.9 |
| Stomach | C16.0-C16.6, C16.8-C16.9 |
| Testicular | C62.0-C62.1, C62.9 |
| Thyroid | C73.0 |
| Uterine | C54.0-C54.3, C54.8-C55.0 |

**Appendix 2: Numbers and crude proportions of cancer cases diagnosed via emergency presentation in 2006-2015**

| **Variable** | **Total number of cases** | **Emergency presentations, n (%)** | |
| --- | --- | --- | --- |
| All cases | 2641428 | 559254 | (21.2) |
| Sex |  |  |  |
| Female | 1299813 | 264377 | (20.3) |
| Male | 1341615 | 294877 | (22.0) |
| Age group (years) |  |  |  |
| 25-49 | 264006 | 32561 | (12.3) |
| 50-59 | 360497 | 49881 | (13.8) |
| 60-69 | 678094 | 105475 | (15.6) |
| 70-79 | 739971 | 159124 | (21.5) |
| 80+ | 598860 | 212213 | (35.4) |
| Deprivation |  |  |  |
| 1 (Least) | 553813 | 95181 | (17.2) |
| 2 | 576697 | 110723 | (19.2) |
| 3 | 552668 | 115834 | (21.0) |
| 4 | 508407 | 119443 | (23.5) |
| 5 (Most) | 449843 | 118073 | (26.2) |
| Cancer (in descending order of % emergency presentation) |  |  |  |
| Brain | 37636 | 22361 | (59.4) |
| ALL | 2158 | 1251 | (58.0) |
| CUP | 80712 | 45366 | (56.2) |
| AML | 23586 | 12283 | (52.1) |
| Pancreatic | 310 | 149 | (48.1) |
| Gallbladder | 75036 | 35139 | (46.8) |
| Small intestinal | 6747 | 3127 | (46.3) |
| Liver | 10492 | 4746 | (45.2) |
| Lung | 23980 | 9588 | (40.0) |
| Mesothelioma | 354608 | 128938 | (36.4) |
| Multiple myeloma | 42669 | 14349 | (33.6) |
| Stomach | 22787 | 7597 | (33.3) |
| CML | 59473 | 19000 | (31.9) |
| Colon | 5733 | 1741 | (30.4) |
| Ovarian | 219118 | 65092 | (29.7) |
| NHL | 60290 | 17286 | (28.7) |
| Renal | 104740 | 27001 | (25.8) |
| Intracranial endocrine | 74138 | 17586 | (23.7) |
| Oesophageal | 70260 | 14505 | (20.6) |
| Sarcoma (soft tissue) | 19096 | 3781 | (19.8) |
| CLL | 29482 | 5694 | (19.3) |
| Bladder | 87869 | 15989 | (18.2) |
| HL | 12331 | 2015 | (16.3) |
| Anal | 9765 | 1230 | (12.6) |
| Rectal | 115160 | 15794 | (13.7) |
| Cervical | 24625 | 2565 | (10.4) |
| Laryngeal | 18407 | 1904 | (10.3) |
| Testicular | 16263 | 1456 | (9.0) |
| Prostate | 366154 | 31485 | (8.6) |
| Uterine | 69510 | 5528 | (8.0) |
| Oropharyngeal | 22405 | 1493 | (6.7) |
| Thyroid | 18695 | 1221 | (6.5) |
| Oral | 23598 | 1321 | (5.6) |
| Breast | 424798 | 18199 | (4.3) |
| Melanoma | 108797 | 2474 | (2.3) |
| Year |  |  |  |
| 2006 | 237799 | 56624 | (23.8) |
| 2007 | 240520 | 54702 | (22.7) |
| 2008 | 251352 | 55920 | (22.2) |
| 2009 | 257463 | 56353 | (21.9) |
| 2010 | 260732 | 54976 | (21.1) |
| 2011 | 266498 | 55966 | (21.0) |
| 2012 | 274291 | 57150 | (20.8) |
| 2013 | 283452 | 57336 | (20.2) |
| 2014 | 284661 | 55710 | (19.6) |
| 2015 | 284660 | 54517 | (19.2) |

ALL = Acute Lymphoblastic Leukaemia; AML = Acute Myeloid Leukaemia; CLL = Chronic Lymphocytic Leukaemia; CML = Chronic Myeloid Leukaemia; CUP = Cancer of Unknown Primary; HL = Hodgkin Lymphoma; NHL = Non-Hodgkin Lymphoma

**Appendix 3: Descriptive statistics on sex, age, deprivation, and cancer site among cancer cases diagnosed every three years from 2006 to 2015**

| **Variable** | **2006** | | **2009** | | **2012** | | **2015** | |
| --- | --- | --- | --- | --- | --- | --- | --- | --- |
| All cases | 237799 | (100.0) | 257463 | (100.0) | 274291 | (100.0) | 284660 | (100.0) |
| Sex |  |  |  |  |  |  |  |  |
| Female | 118042 | (49.6) | 126088 | (49.0) | 134310 | (49.0) | 139674 | (49.1) |
| Male | 119757 | (50.4) | 131375 | (51.0) | 139981 | (51.0) | 144986 | (50.9) |
| Age-group (years) |  |  |  |  |  |  |  |  |
| 25-49 | 23830 | (10.0) | 26330 | (10.2) | 27519 | (10.0) | 27697 | (9.7) |
| 50-59 | 34370 | (14.5) | 34597 | (13.4) | 36277 | (13.2) | 39638 | (13.9) |
| 60-69 | 57578 | (24.2) | 66390 | (25.8) | 71710 | (26.1) | 73477 | (25.8) |
| 70-79 | 67976 | (28.6) | 72120 | (28.0) | 75782 | (27.6) | 80389 | (28.2) |
| 80+ | 54045 | (22.7) | 58026 | (22.5) | 63003 | (23.0) | 63459 | (22.3) |
| Deprivation |  |  |  |  |  |  |  |  |
| 1 (Least) | 46686 | (19.6) | 51383 | (20.0) | 58992 | (21.5) | 62121 | (21.8) |
| 2 | 50652 | (21.3) | 56204 | (21.8) | 60229 | (22.0) | 62764 | (22.0) |
| 3 | 50785 | (21.4) | 53939 | (21.0) | 57028 | (20.8) | 59232 | (20.8) |
| 4 | 47333 | (19.9) | 50898 | (19.8) | 52066 | (19.0) | 53785 | (18.9) |
| 5 (Most) | 42343 | (17.8) | 45039 | (17.5) | 45976 | (16.8) | 46758 | (16.4) |
| Cancer site (in descending order of % emergency presentation*) |  |  |  |  |  |  |  |  |
| Brain | 3379 | (1.4) | 3775 | (1.5) | 3821 | (1.4) | 4040 | (1.4) |
| ALL | 215 | (0.1) | 203 | (0.1) | 214 | (0.1) | 241 | (0.1) |
| CUP | 9457 | (4.0) | 8140 | (3.2) | 7789 | (2.8) | 7089 | (2.5) |
| AML | 2093 | (0.9) | 2308 | (0.9) | 2440 | (0.9) | 2622 | (0.9) |
| Intracranial endocrine | 19 | (0.0) | 24 | (0.0) | 41 | (0.0) | 31 | (0.0) |
| Pancreatic | 6720 | (2.8) | 7233 | (2.8) | 7817 | (2.8) | 8314 | (2.9) |
| Gallbladder | 526 | (0.2) | 586 | (0.2) | 702 | (0.3) | 875 | (0.3) |
| Small intestinal | 748 | (0.3) | 967 | (0.4) | 1107 | (0.4) | 1336 | (0.5) |
| Liver | 1620 | (0.7) | 2084 | (0.8) | 2676 | (1.0) | 3010 | (1.1) |
| Lung | 32687 | (13.7) | 34092 | (13.2) | 37233 | (13.6) | 37621 | (13.2) |
| Multiple myeloma | 3572 | (1.5) | 4202 | (1.6) | 4445 | (1.6) | 4628 | (1.6) |
| Mesothelioma | 2100 | (0.9) | 2258 | (0.9) | 2474 | (0.9) | 2342 | (0.8) |
| Stomach | 6347 | (2.7) | 6202 | (2.4) | 5763 | (2.1) | 5489 | (1.9) |
| CML | 536 | (0.2) | 584 | (0.2) | 564 | (0.2) | 588 | (0.2) |
| Colon | 19417 | (8.2) | 21625 | (8.4) | 22865 | (8.3) | 23324 | (8.2) |
| Ovarian | 5796 | (2.4) | 6040 | (2.3) | 6109 | (2.2) | 6091 | (2.1) |
| NHL | 8852 | (3.7) | 10237 | (4.0) | 11092 | (4.0) | 11470 | (4.0) |
| Renal | 5777 | (2.4) | 6686 | (2.6) | 7721 | (2.8) | 8909 | (3.1) |
| Oesophageal | 6450 | (2.7) | 6768 | (2.6) | 7389 | (2.7) | 7605 | (2.7) |
| Sarcoma (connect and soft) | 1527 | (0.6) | 1860 | (0.7) | 2102 | (0.8) | 2143 | (0.8) |
| CLL | 2493 | (1.0) | 3099 | (1.2) | 3075 | (1.1) | 3245 | (1.1) |
| Bladder | 8492 | (3.6) | 8884 | (3.5) | 9038 | (3.3) | 8490 | (3.0) |
| HL | 1063 | (0.4) | 1235 | (0.5) | 1236 | (0.5) | 1424 | (0.5) |
| Anal | 766 | (0.3) | 912 | (0.4) | 1070 | (0.4) | 1260 | (0.4) |
| Rectal | 11204 | (4.7) | 11692 | (4.5) | 12223 | (4.5) | 11222 | (3.9) |
| Cervical | 2337 | (1.0) | 2722 | (1.1) | 2491 | (0.9) | 2444 | (0.9) |
| Laryngeal | 1763 | (0.7) | 1831 | (0.7) | 1921 | (0.7) | 1882 | (0.7) |
| Testicular | 1524 | (0.6) | 1638 | (0.6) | 1657 | (0.6) | 1697 | (0.6) |
| Prostate | 31820 | (13.4) | 36246 | (14.1) | 38082 | (13.9) | 40310 | (14.2) |
| Uterine | 6044 | (2.5) | 6589 | (2.6) | 7315 | (2.7) | 7449 | (2.6) |
| Thyroid | 1618 | (0.7) | 1965 | (0.8) | 2515 | (0.9) | 2879 | (1.0) |
| Oropharyngeal | 1274 | (0.5) | 1580 | (0.6) | 2107 | (0.8) | 2679 | (0.9) |
| Oral | 1926 | (0.8) | 2280 | (0.9) | 2577 | (0.9) | 2635 | (0.9) |
| Breast | 38934 | (16.4) | 40901 | (15.9) | 43291 | (15.8) | 46078 | (16.2) |
| Melanoma | 8703 | (3.7) | 10015 | (3.9) | 11329 | (4.1) | 13198 | (4.6) |

*See Appendix 2.

ALL = Acute Lymphoblastic Leukaemia; AML = Acute Myeloid Leukaemia; CLL = Chronic Lymphocytic Leukaemia; CML = Chronic Myeloid Leukaemia; CUP = Cancer of Unknown Primary; HL = Hodgkin Lymphoma; NHL = Non-Hodgkin Lymphoma
